# Feature-Level Fusion of Surface Electromyography and Mechanomyography Signals for MVC-Normalized Shoulder Abduction Force-Level Classification in Healthy Adults

**DOI:** 10.3390/s26144351

**Published:** 2026-07-09

**Authors:** Chuangan Zhou, Yuzhu Gao, Xingyue Gou, Junyu Yao, Qinwei Wu, Dong Cao, Xiaohua He, Jun Yi

**Affiliations:** 1 School of Medical Informatics Engineering, Guangzhou University of Chinese Medicine, Guangzhou 510006, China; 2 School of Medical Information Engineering, Guangdong Pharmaceutical University, Guangzhou 510006, China

**Keywords:** surface electromyography, mechanomyography, MVC-normalized force level, shoulder abduction, feature-level fusion, wearable sensors, machine learning

## Abstract

Background: Accurate recognition of upper-limb force levels is important for wearable movement monitoring and rehabilitation engineering, yet the value of combining surface electromyography (sEMG) and mechanomyography (MMG) for shoulder force classification remains incompletely characterized. Methods: Ten healthy adults performed right shoulder abduction at four maximum voluntary contraction (MVC)-normalized force levels of approximately 10%, 30%, 60%, and 90% MVC. Signals were synchronously collected from the middle deltoid at 1000 Hz and segmented using 500 ms windows with a 150 ms stride. The evaluated classifiers were logistic regression (LR), k-nearest neighbors (KNN), decision tree (DT), support vector machine with a radial basis function kernel (SVM-RBF), random forest (RF), extremely randomized trees (ET), histogram-based gradient boosting decision tree (HGBDT), and multi-layer perceptron (MLP). Models were evaluated using group-aware five-fold cross-validation at the action-trial level. Results: The dataset contained 12,231 windows from 30 action-trial groups. HGBDT achieved the best performance, with an accuracy of 0.904±0.023, macro-F1 score of 0.911±0.018, quadratic weighted Cohen’s kappa of 0.910±0.038, and mean absolute grade error of 0.137±0.042. Fusion increased macro-F1 from 0.821±0.030 for sEMG-only and 0.771±0.015 for MMG-only to 0.911±0.018. Conclusions: These internally validated findings support the complementary value of sEMG and MMG for MVC-normalized shoulder force-level classification in healthy adults. Subject-independent and patient-level validation is required before clinical rehabilitation use.

## 1. Introduction

Objective quantification of upper-limb force production is increasingly relevant to wearable movement monitoring, rehabilitation engineering, and individualized motor training, because force-level changes cannot always be captured by visual observation or coarse ordinal assessment alone [1,2]. Manual muscle testing and related ordinal scales are simple and clinically accessible, but their limited score resolution and potential inter-rater variability make it difficult to identify small changes in force generation during repeated or high-score movements [3,4]. Wearable biosignals provide quantitative measurements of muscle electrical activation, mechanical vibration, and movement-related responses during standardized tasks [5,6]. Surface electromyography (sEMG) has been widely used to characterize muscle activation and motor-unit behavior, although its amplitude and spectral features can be influenced by electrode–skin impedance, crosstalk, motion artifacts, and preprocessing choices [7,8,9,10]. Mechanomyography (MMG) provides a complementary mechanical view of muscle contraction by capturing surface vibration, contractile oscillation, and load-related changes that are not directly represented in sEMG [5,11,12].

Recent work in multimodal wearable sensing suggests that combining neuromuscular, mechanical, and movement-derived information can improve sensor-based movement and fatigue assessment [13,14,15]. In shoulder and elbow motion estimation, biosignal fusion improved the coefficient of determination by 0.041–0.053 compared with MMG-only inputs [13]. A manual-lifting study also reported high fatigue-classification accuracy when sEMG and MMG information was jointly modeled [16]. Experimental studies using MVC and submaximal loading further support MMG as a mechanical counterpart to sEMG in force-related tasks [11,12]. However, previous work has mainly addressed joint acceleration, fatigue, gesture recognition, or load-lifting behavior. Evidence remains limited on whether sEMG and MMG provide complementary information for discrete MVC-normalized shoulder-abduction force levels, particularly when single-modality and fused feature sets are compared directly.

We hypothesized that the feature-level fusion of sEMG and MMG would improve four-level MVC-normalized shoulder-abduction classification and reduce ordinal prediction error compared with either modality alone. To test this hypothesis, we benchmarked eight supervised classifiers and compared sEMG-only, MMG-only, directly concatenated, and correlation-augmented fusion feature sets. Group-aware cross-validation at the action-trial level was used to reduce leakage between overlapping windows, and class-specific errors were examined to characterize the limits of force-level separability [17,18].

## 2. Materials and Methods

**Figure 1:**
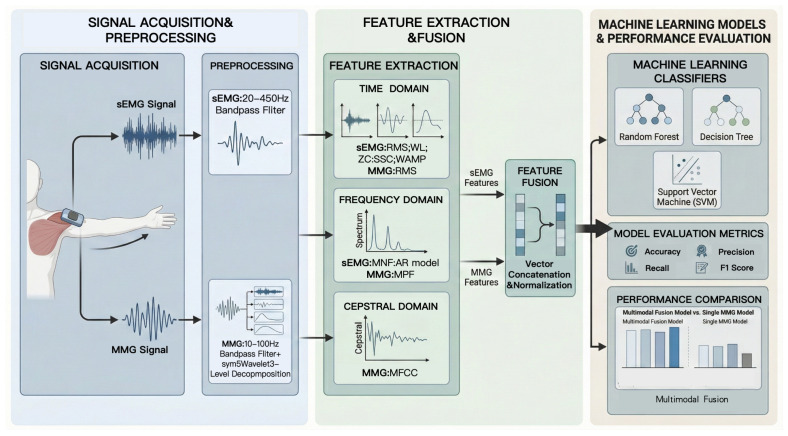
Overall workflow for multimodal signal acquisition, preprocessing, feature extraction, feature-level fusion, machine-learning classification, and performance evaluation.

This healthy-volunteer study evaluated whether the feature-level fusion of surface electromyography (sEMG) and mechanomyography (MMG) signals could improve the classification of maximum voluntary contraction (MVC)-normalized shoulder-abduction force levels. Shoulder abduction was selected, because it is relevant to upper-limb function and rehabilitation training and permits controlled loading across relative force levels. The middle deltoid was selected as a major superficial agonist during shoulder abduction and as an accessible site for colocated noninvasive sEMG and MMG acquisition [19,20]. The overall workflow included multimodal signal acquisition, preprocessing, sliding-window feature extraction, feature-level fusion, supervised classification, group-aware validation at the action-trial level, and feature-set ablation (Figure 1).

### 2.1. Participants

Ten healthy adults were recruited, including six males and four females. All participants were right-handed. Their age, height, and body weight were 24.8±1.9 years, 170.6±8.7 cm, and 68.9±6.4 kg, respectively. None reported upper-limb neuromuscular or musculoskeletal disorders. Participants were instructed to avoid fitness training, resistance exercise, and other activities that imposed substantial upper-limb loading during the week before testing. All participants were informed of the experimental procedure and signed informed consent forms before data collection. Because the experiment involved healthy adults, the labels were interpreted as MVC-normalized force-level categories rather than clinically assigned manual muscle testing (MMT) grades.

### 2.2. Experimental Protocol and Force-Level Labeling

Participants sat upright with the trunk maintained in a neutral position, and the right upper limb was used as the test side. Before formal acquisition, each participant practiced the shoulder-abduction task under the supervision of a rehabilitation physician. Each repetition began with the upper limb in the neutral position. The participant then abducted the arm while holding the assigned adjustable dumbbell and maintained the abducted posture for 2 s. Each movement was repeated 10 times, and each participant completed three sets. A 2 min rest interval was provided between sets to reduce fatigue-related changes in the signals.

Before formal data collection, each participant completed five shoulder-abduction trials at the maximum load achievable with the adjustable dumbbell. The maximal abducted posture was maintained for 2 s in each trial, and the mean load was used as the individual MVC reference. Target dumbbell loads were then set at approximately 10%, 30%, 60%, and 90% of this reference. These conditions were assigned to Labels 2, 3, 4, and 5, respectively (Table 1). The labels therefore represent four MVC-normalized external-load conditions under a controlled healthy-volunteer protocol and should not be interpreted as direct clinical MMT scores.

### 2.3. Signal Acquisition and Preprocessing

sEMG and MMG signals were recorded using a custom-developed wearable acquisition device. The sEMG channel used dry bipolar surface electrodes with a center-to-center spacing of 20 mm, an input range of ±5 mV, a gain of 1000, and input-referred noise of no more than 5 μV RMS. The MMG channel incorporated an ADXL355 three-axis digital microelectromechanical systems (MEMS) accelerometer (Analog Devices, Inc. Wilmington, MA, USA). According to the manufacturer’s specifications, the ADXL355 supports user-selectable ±2 g, ±4 g, and ±8 g ranges, uses a 20-bit analog-to-digital converter, and has a typical sensitivity of 256,000 LSB/g in the ±2 g range. In the present protocol, the sEMG and MMG channels were synchronously sampled from the middle deltoid at 1000 Hz using a shared timestamp. The signals were transmitted to a laptop via Bluetooth and subsequently exported for feature extraction and modeling.

**Figure 2:**
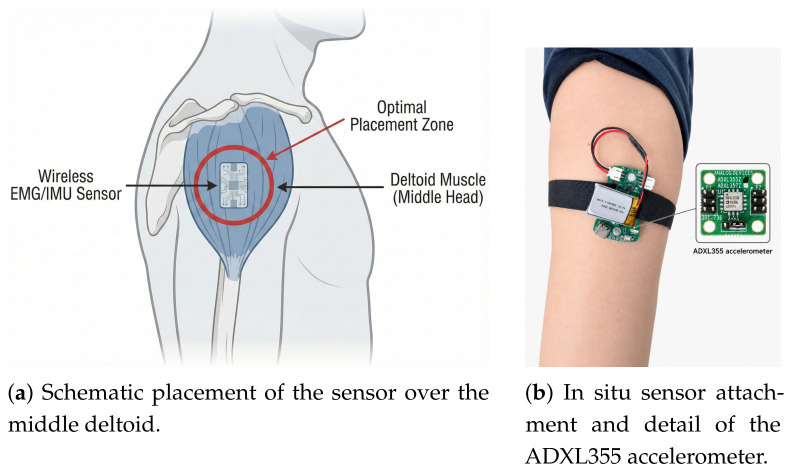
Sensor placement and hardware used for shoulder-abduction signal acquisition. The schematic shows the recommended middle-deltoid placement region, and the de-identified photograph shows the practical attachment of the custom-developed wearable acquisition device and its ADXL355 accelerometer.

Before sensor placement, the skin surface was cleaned with alcohol wipes to reduce skin impedance. Sensor placement followed the general SENIAM recommendations for the middle deltoid [20]. The sensor was placed near the midpoint between the acromion and the lateral epicondyle of the humerus, and its longitudinal axis was aligned with the muscle-fiber direction (Figure 2).

**Figure 3:**
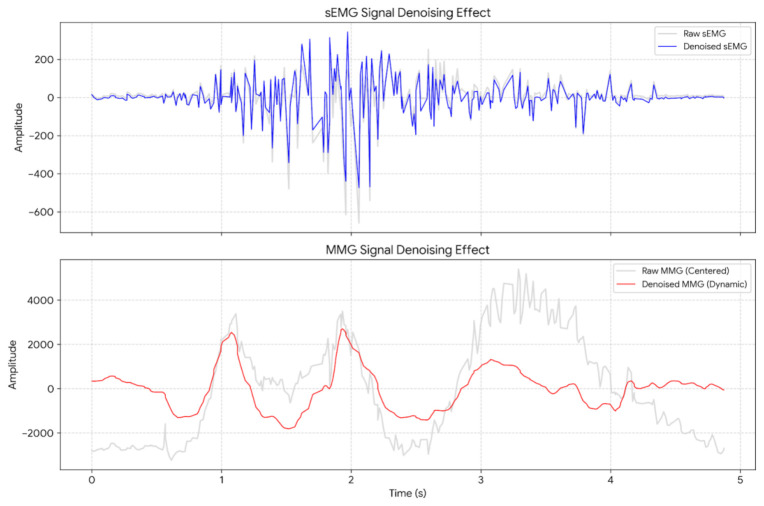
Representative preprocessing results for sEMG and MMG signals. The upper panel compares raw sEMG with the signal after 20–450 Hz band-pass filtering. The lower panel compares centered raw MMG with MMG after 10–100 Hz band-pass filtering and sym5 wavelet soft-threshold denoising.

The raw signals were preprocessed before feature extraction. For MMG, a fourth-order Butterworth band-pass filter with cutoff frequencies of 10 and 100 Hz was applied. Wavelet denoising was then performed using the sym5 wavelet basis with level-3 decomposition and soft-thresholding to reduce motion artifacts while retaining mechanical vibration information. For sEMG, a fourth-order Butterworth band-pass filter with cutoff frequencies of 20 and 450 Hz was used. No separate 50-Hz power-line notch filter was applied. Figure 3 shows representative preprocessing results for the sEMG and MMG signals.

### 2.4. Feature Extraction and Feature-Level Fusion

Preprocessed continuous signals were segmented using a sliding-window strategy. The window length was set to 500 ms, and the stride was set to 150 ms. For each window, the predefined 26-feature set was retained for the main analysis, including 10 sEMG-derived features, 15 MMG-derived features, and one correlation-based fusion feature (Table 2). Three inertial measurement unit (IMU)-derived kinematic features in the feature table were not included, because the present study focused on sEMG-MMG feature-level fusion.

The 10 sEMG-derived features included time-domain energy and complexity features, a frequency-domain feature, and model-based features: root mean square (EMG_RMS), waveform length (EMG_WL), zero crossing (EMG_ZC), slope sign change (EMG_SSC), Willison amplitude (EMG_WAMP), mean frequency (EMG_MNF), and fourth-order autoregressive coefficients (EMG_AR1–EMG_AR4). The 15 MMG-derived features included MMG root mean square (MMG_RMS_Mech), kurtosis (MMG_Kurtosis), mean power frequency (MMG_MPF), and 12 Mel-frequency cepstral coefficients (MMG_MFCC_1–MMG_MFCC_12). The additional Fusion_Corr_MA feature was retained from the predefined feature table as a window-level correlation-based feature designed to represent local sEMG-MMG amplitude coupling.

Feature-level fusion was implemented by concatenating the sEMG-derived, MMG-derived, and correlation-based features into a unified feature vector before model training. Let $F_{\mathrm{sEMG}}\in R^{10}$ denote the sEMG feature vector and $F_{\mathrm{MMG}}\in R^{15}$ denote the MMG feature vector. The predefined 26-feature fusion vector was defined as

(1) $$F_{\mathrm{fusion}26}=\left[F_{\mathrm{sEMG}},F_{\mathrm{MMG}},F_{\mathrm{CorrMA}}\right]\in R^{26},$$

where $F_{\mathrm{CorrMA}}$ represents the Fusion_Corr_MA feature. This window-level electromechanical coupling feature was calculated as the Pearson correlation between the time-aligned moving-average amplitude sequences of rectified sEMG and MMG:

(2) $$F_{\mathrm{CorrMA}}=\frac{\sum_{i=1}^{n}\left(u_{i}-\bar{u}\right)\left(v_{i}-\bar{v}\right)}{\sqrt{\sum_{i=1}^{n}{\left(u_{i}-\bar{u}\right)}^{2}}\sqrt{\sum_{i=1}^{n}{\left(v_{i}-\bar{v}\right)}^{2}}},$$

where $u_{i}=\mathrm{MA}(|x_{\mathrm{sEMG},i}\left|\right)$ and $v_{i}=\mathrm{MA}(|x_{\mathrm{MMG},i}\left|\right)$ denote the paired moving-average amplitude sequences within the same 500 ms analysis window. The feature was intended to summarize local covariation between electrical activation and mechanical vibration. For scale-sensitive models, including LR, KNN, SVM-RBF, and MLP, z-score standardization was fitted only on the training folds and then applied to the corresponding validation folds. Tree-based models were trained using the extracted feature values directly.

### 2.5. Feature-Set Ablation Design

To examine whether the performance gain was attributable to multimodal fusion rather than a single signal source, four feature sets were compared in the ablation analysis (Table 3). The main feature set was the predefined 26-feature fusion set used in the main analysis. The 25-feature sEMG+MMG set was additionally analyzed to examine whether the correlation-based fusion feature changed model performance beyond direct concatenation of sEMG and MMG features.

### 2.6. Classification Models

Eight representative supervised classifiers were evaluated to compare the performance of the feature-level fusion strategy: logistic regression (LR), k-nearest neighbors (KNN), decision tree (DT), support vector machine with radial basis function kernel (SVM-RBF), random forest (RF), extremely randomized trees (ET), histogram-based gradient boosting decision tree (HGBDT), and multi-layer perceptron (MLP). These models were selected to cover linear, distance-based, single-tree, margin-based, bagging ensemble, randomized-tree ensemble, boosting ensemble, and shallow neural-network classifiers.

Hyperparameters were selected empirically through repeated preliminary comparisons before the formal evaluation. The selected parameter set was then held fixed across all five cross-validation folds.

LR was implemented with L2 regularization, C=1.0, class-balanced weighting, and a maximum of 5000 iterations. KNN used seven neighbors with distance weighting. DT used the Gini criterion with class-balanced weighting. SVM-RBF used C=10, gamma set to “scale”, and class-balanced weighting. RF and ET each used 100 trees, class-balanced weighting, and unrestricted maximum depth. HGBDT was configured with 120 boosting iterations, a learning rate of 0.05, 31 maximum leaf nodes, L2 regularization of 0.01, and early stopping. MLP used two hidden layers with 64 and 32 neurons, rectified linear unit activation, Adam optimization, L2 regularization coefficient of 0.001, adaptive learning rate, and early stopping. The random seed was set to 42 for reproducibility when applicable.

### 2.7. Validation Strategy and Performance Metrics

Because the feature samples were generated from overlapping sliding windows, random row-level splitting could overestimate the classification performance by placing highly correlated neighboring windows in both training and validation sets. Therefore, group-aware cross-validation at the action-trial level was used as the primary validation strategy. The action-trial identifier was retained from the analysis feature table, and all windows from the same action trial were assigned to the same fold. Five folds were used. This design evaluated recognition of unseen action trials and reduced leakage between neighboring windows. It did not ensure that all trials from one participant were restricted to a single fold.

For each fold, models were trained on the training action-trial groups and evaluated on unseen action-trial groups. The performance was summarized using accuracy, balanced accuracy, macro-precision, macro-recall, macro-F1, weighted-F1, linear weighted Cohen’s kappa, quadratic weighted Cohen’s kappa (QWK), mean absolute grade error (MAE), and within-one-grade accuracy. Weighted kappa and MAE were included, because the target labels represented ordered force-level categories. Within-one-grade accuracy was defined as the proportion of samples for which the absolute difference between the predicted and true labels was no greater than 1.

## 3. Results

### 3.1. Analysis Dataset and Label Distribution

After sliding-window segmentation and feature extraction, the analysis feature table contained 12,231 window-level samples from 30 action-trial groups. Four MVC-normalized force-level labels were included. Label 2 accounted for 2825 samples (23.10%), Label 3 for 2387 samples (19.52%), Label 4 for 3403 samples (27.82%), and Label 5 for 3616 samples (29.56%). The dataset structure is summarized in Table 4.

### 3.2. Comparison of Eight Classifiers Using the Predefined 26-Feature Fusion Set

**Figure 4:**
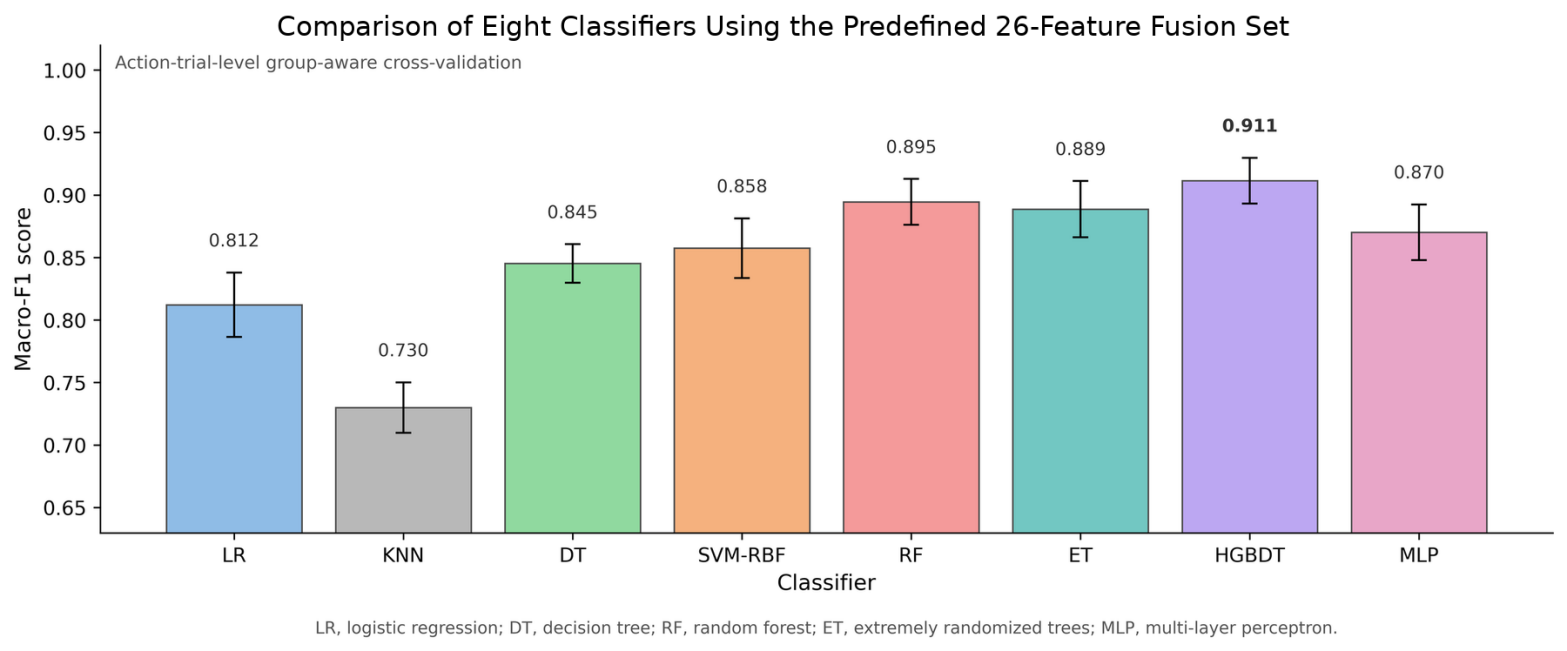
Comparison of eight classifiers using the predefined 26-feature fusion set under group-aware cross-validation at the action-trial level. Bars indicate mean macro-F1 scores, and error bars indicate standard deviations across folds. LR, logistic regression; KNN, k-nearest neighbors; DT, decision tree; SVM-RBF, support vector machine with radial basis function kernel; RF, random forest; ET, extremely randomized trees; HGBDT, histogram-based gradient boosting decision tree; MLP, multi-layer perceptron.

Using the predefined 26-feature fusion set, HGBDT achieved the best overall performance among the eight evaluated classifiers (Table 5 and Figure 4). Under group-aware cross-validation at the action-trial level, HGBDT achieved an accuracy of 0.904±0.023, balanced accuracy of 0.912±0.017, macro-F1 score of 0.911±0.018, QWK of 0.910±0.038, and MAE of 0.137±0.042. RF and ET achieved the next best performances, with macro-F1 scores of 0.895±0.018 and 0.889±0.023, respectively. MLP achieved a macro-F1 score of 0.870±0.022, followed by SVM-RBF, DT, LR, and KNN.

The results suggest that the nonlinear ensemble models were more suitable for capturing the electromechanical feature interactions between sEMG and MMG signals than linear or distance-based baselines. HGBDT was therefore selected as the main classifier for subsequent feature-set ablation analysis and confusion-matrix evaluation.

### 3.3. Ablation Analysis of Single-Modality and Fusion Feature Sets

**Figure 5:**
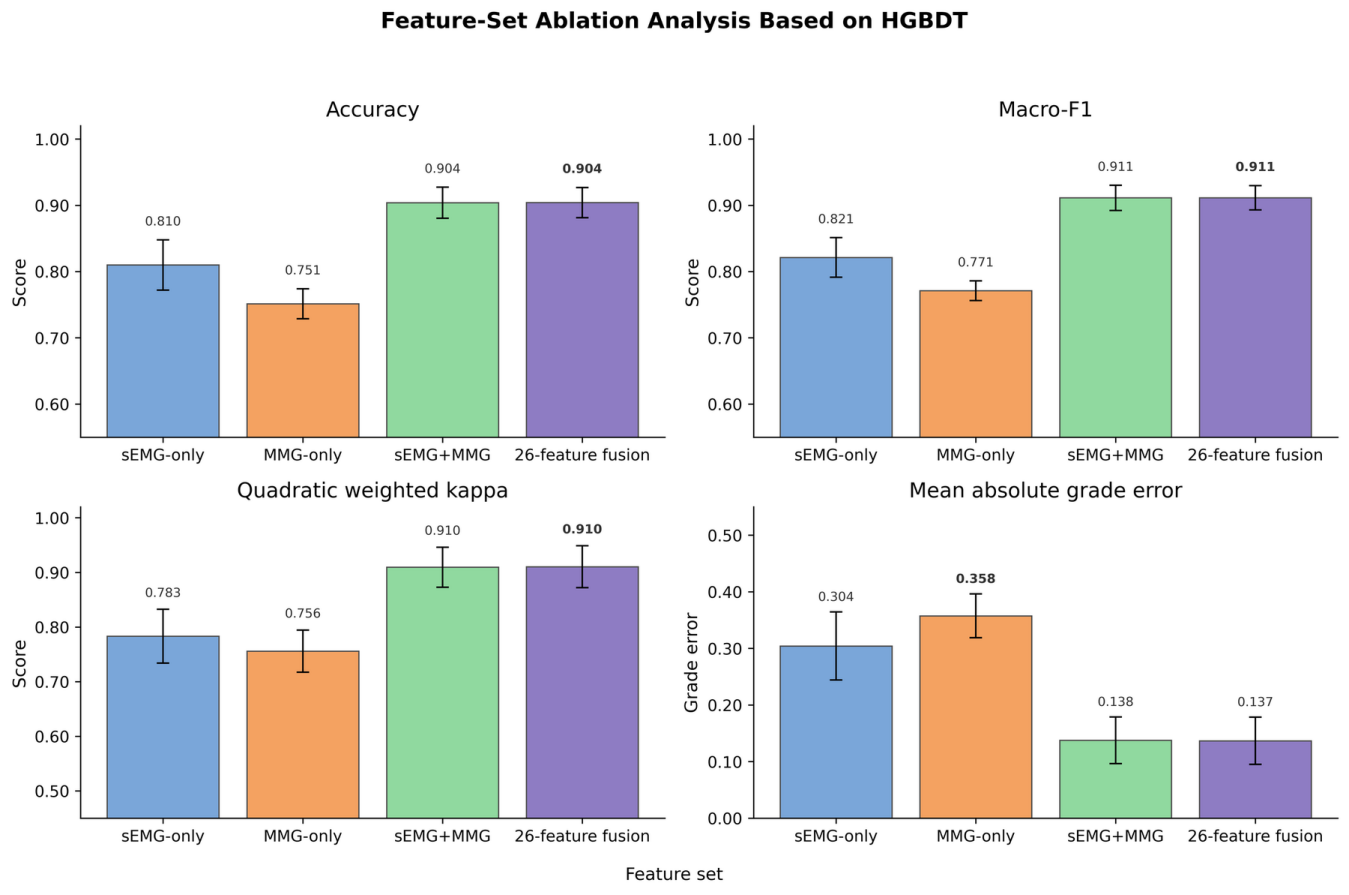
Ablation analysis of single-modality and fusion feature sets using HGBDT under group-aware cross-validation at the action-trial level. Bars indicate mean performance values, and error bars indicate standard deviations across folds.

The ablation analysis showed that multimodal feature fusion improved the force-level classification compared with either single-modality input (Table 6 and Figure 5). Using HGBDT as the main classifier, the sEMG-only feature set achieved a macro-F1 score of 0.821±0.030, whereas the MMG-only feature set achieved a macro-F1 score of 0.771±0.015. Direct concatenation of sEMG and MMG features increased the macro-F1 score to 0.911±0.019. The predefined 26-feature fusion set, which additionally included Fusion_Corr_MA, achieved a macro-F1 score of 0.911±0.018, a QWK of 0.910±0.038, and an MAE of 0.137±0.042.

The improvement in macro-F1 of the predefined 26-feature fusion set was approximately 0.090 over the sEMG-only feature set and approximately 0.140 over the MMG-only feature set. The 25-feature sEMG+MMG set and the predefined 26-feature set showed nearly identical macro-F1 values, indicating that the main performance gain was associated to the complementary integration of sEMG-derived and MMG-derived features rather than to the correlation-based fusion feature alone.

### 3.4. Confusion Matrix and Per-Class Performance of the 26-Feature HGBDT Model

**Figure 6:**
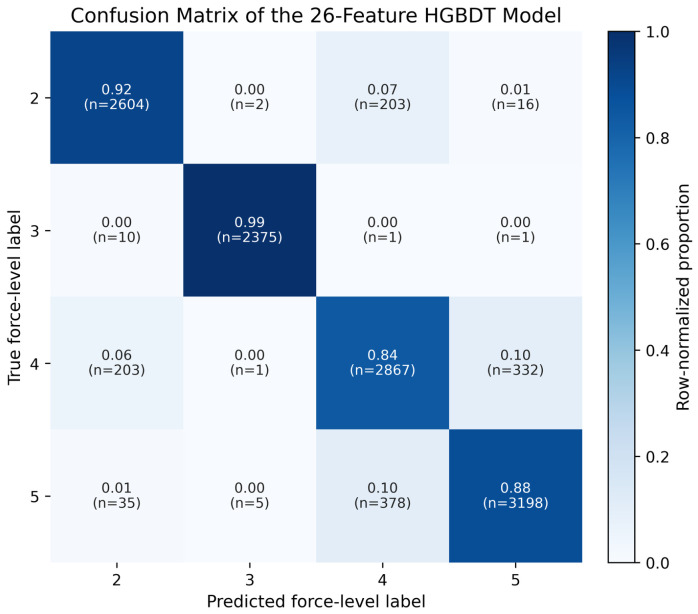
Normalized confusion matrix of the HGBDT model using the predefined 26-feature fusion set. Values represent row-normalized proportions, and the number in parentheses indicates the corresponding sample count.

Out-of-fold predictions from the 26-feature HGBDT model were used to generate the confusion matrix and per-class performance results (Figure 6 and Table 7). The overall out-of-fold accuracy was 0.903, the balanced accuracy was 0.911, the macro-F1 score was 0.911, the QWK was 0.910, and the MAE was 0.139. The within-one-grade accuracy was 0.962, indicating that most prediction errors were within one force-level category.

Label 3 showed the highest classification stability, with a precision of 0.997, a recall of 0.995, and an F1 score of 0.996. Label 4 showed the lowest F1 score among the four labels (0.837), reflecting higher overlap between moderate-to-high and high force-level signal patterns. In the normalized confusion matrix, 9.84% of Label 4 samples were predicted as Label 5, and 10.45% of Label 5 samples were predicted as Label 4. In addition, 7.22% of Label 2 samples were predicted as Label 4, and 5.99% of Label 4 samples were predicted as Label 2. These findings suggest that the force-level separability was not uniform across labels, with the main classification difficulty concentrated around intermediate-to-high force levels and selected cross-level overlaps.

### 3.5. Feature-Importance Analysis

**Figure 7:**
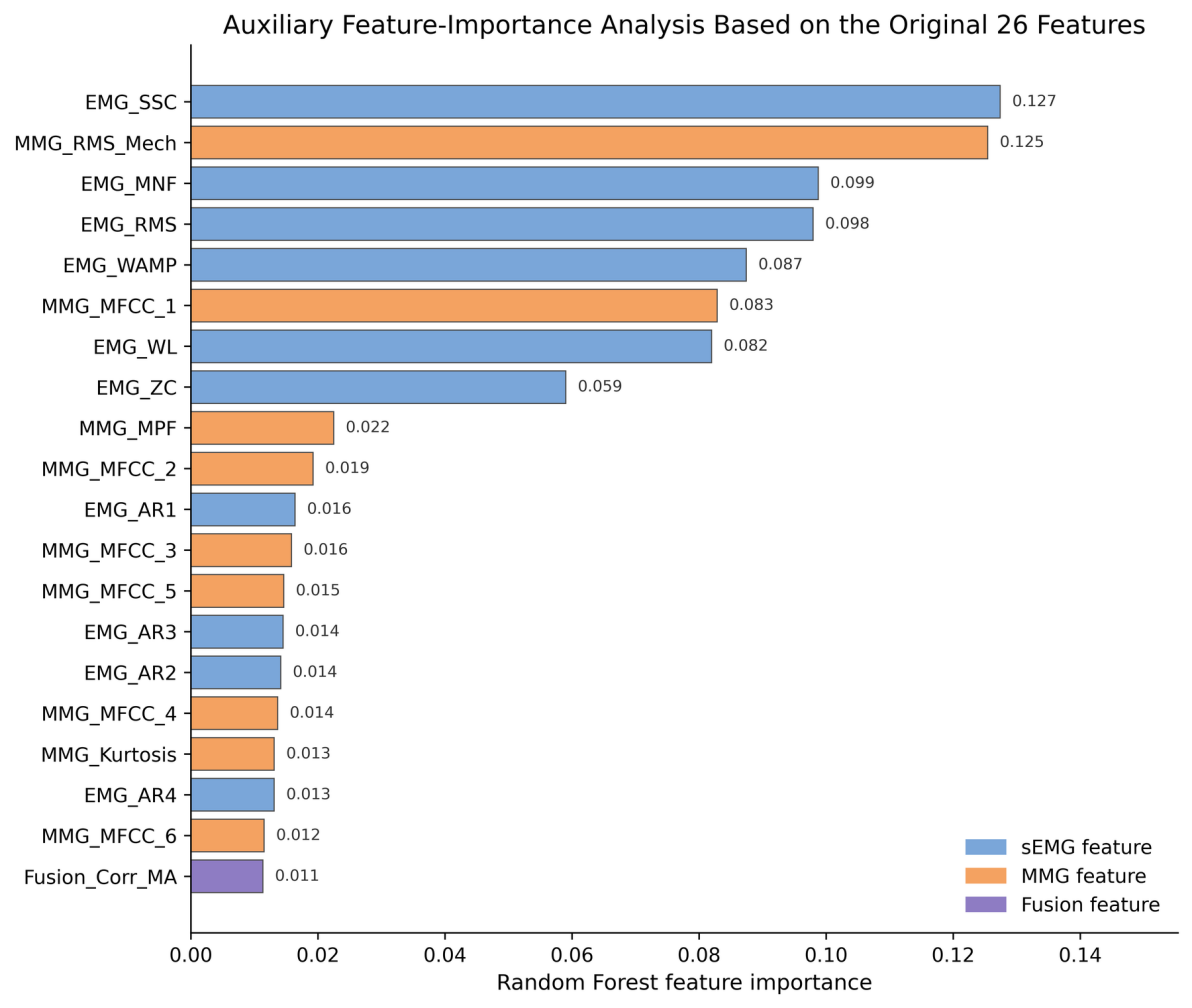
RF-based feature-importance analysis for the predefined 26-feature set. The analysis was used to explore feature contribution rather than to determine the best classifier.

An RF-based feature-importance analysis was performed to explore the relative contribution of the predefined 26 features (Figure 7). This analysis was used only for feature interpretation and was not used to select the best classifier. The highest-ranked features included EMG_SSC (importance, 0.127), MMG_RMS_Mech (0.125), EMG_MNF (0.099), EMG_RMS (0.098), EMG_WAMP (0.087), MMG_MFCC_1 (0.083), and EMG_WL (0.082). These results indicate that both sEMG-derived and MMG-derived features contributed to force-level classification. The correlation-based Fusion_Corr_MA feature contributed modestly (0.011), further supporting that the main performance gain was primarily driven by the combined use of sEMG-derived and MMG-derived information.

## 4. Discussion

### 4.1. Principal Findings

The principal finding was that sEMG-MMG feature fusion outperformed either modality alone for four-level MVC-normalized shoulder-abduction classification under action-trial-level validation. The ablation results indicate that the improvement arose mainly from combining electrical and mechanical feature families [7,11]. Adding Fusion_Corr_MA to the directly concatenated feature set produced only a marginal numerical change. This feature should therefore be interpreted as an exploratory coupling variable rather than a major source of the observed performance gain.

### 4.2. Relation to Previous Wearable-Sensing Studies

Related studies have addressed different but complementary sensing targets. Correa et al. examined EMG and accelerometer-derived MMG responses during upper-limb contractions and load lifting, focusing on the signal sensitivity across loading conditions rather than force-level classification [11]. Bai et al. fused biosignals to estimate continuous shoulder and elbow rotational acceleration [13], whereas Wang et al. combined sEMG and joint-motion data for regression-based shoulder-strength assessment [21]. In contrast, the present study classifies four ordered MVC-normalized shoulder-abduction force levels and explicitly evaluates sEMG-only, MMG-only, and fused feature sets; because the tasks and validation designs differ, the reported performance values should not be compared directly.

### 4.3. Physiological Interpretation and Error Pattern

The higher performance of the fused feature set is physiologically plausible because sEMG and MMG reflect related but distinct aspects of voluntary muscle contraction [7,11]. sEMG features describe electrical activation, waveform complexity, and frequency-related changes, whereas the MMG features capture muscle-surface vibration and mechanical oscillation [5,8]. EMG_SSC and MMG_RMS_Mech were the two highest-ranked RF features. MMG_RMS_Mech summarizes the overall amplitude of muscle-surface vibration and may vary with fiber contraction, motor-unit recruitment, muscle stiffness, and transmission through superficial tissue. EMG_SSC quantifies the changes in waveform-slope direction and therefore reflects the complexity and frequency-related content of electrical activation. Their joint prominence supports a complementary, rather than competing, interpretation of electrical and mechanical information.

The force-level separability was not uniform. Label 3 had the fewest samples but the highest class-specific performance, making class frequency an unlikely sole explanation for its stability. Under this controlled protocol, the approximately 30% MVC condition may have produced a comparatively consistent electromechanical pattern. In contrast, some 10% MVC windows were classified as 60% MVC. Low-intensity sEMG has a smaller amplitude and may be more susceptible to baseline noise, electrode-contact variation, and inter-individual differences. Accelerometer-derived MMG may also contain soft-tissue vibration and small movement-related components. Moreover, sEMG amplitude does not remain strictly proportional to the external load across contraction intensities. These factors may cause a partial overlap between Labels 2 and 4 in the fused feature space. This interpretation is hypothesis-generating, because the present experiment was not designed to isolate the contribution of each factor [22,23].

The four force levels were inspired by the ordered logic of clinical manual muscle testing, but they were defined operationally as 10%, 30%, 60%, and 90% MVC-normalized loads. With future comparison against clinical scales and validation in patient cohorts, this approach may provide a more objective and repeatedly measurable sensor-assisted complement to ordinal strength assessment. The present labels should not be interpreted as equivalent to physician-assigned MMT grades [3,4].

### 4.4. Strengths, Limitations, and Future Work

A strength of this study is the combined use of an eight-classifier benchmark, feature-set ablation, ordinal metrics, and action-trial-level grouped validation [18,24]. Grouping overlapping windows by action trial reduced an important source of information leakage [17,18]. However, several limitations remain. First, only 10 healthy adults were included. The observed 90.4% accuracy is therefore an internal performance estimate for unseen action trials within this cohort and not evidence that the same accuracy will be maintained in new participants.

Second, demographic information was available for cohort description, but the de-identified window-level feature table used for modeling did not retain explicit participant identifiers. Consequently, trials from the same participant could occur in different folds, and leave-one-subject-out validation could not be performed. Subject-independent generalization requires a dataset that preserves participant-level grouping and an external cohort.

Third, the findings cannot be generalized directly to neurological patients. Weakness may reduce sEMG amplitude and MMG vibration energy, whereas spasticity may increase background activity or involuntary bursts. Abnormal synergies and cocontraction may increase the overlap between force-level distributions, and compensatory recruitment may alter the temporal and spatial patterns captured at the middle deltoid [1,2]. Dynamic and multi-angle rehabilitation movements may introduce additional kinematic and motion-related variability.

Fourth, the labels were not validated against physician-assigned MMT, handheld dynamometry, or standardized upper-limb functional scales [3,21]. Fifth, the analysis used offline feature extraction and model evaluation; so, real-time performance on wearable or embedded hardware remains untested. Finally, no separate power-line notch filter was applied, and the potential influence of residual 50-Hz interference on sEMG features cannot be excluded. Future work should preserve subject identifiers, evaluate independent participants and neurological cohorts, compare predictions with external strength measures, and test dynamic tasks and real-time deployment [6,25,26,27,28,29,30].

## 5. Conclusions

Feature-level fusion of sEMG and MMG improved MVC-normalized shoulder-abduction force-level classification in this healthy-adult cohort. HGBDT achieved the best internal action-trial-level performance among the eight evaluated classifiers, and the ablation analysis attributed the main gain to combining electrical and mechanical feature families. These findings support the complementary value of sEMG and MMG for sensor-based force assessment under controlled conditions. Subject-independent, patient-level, and external validation is required before clinical rehabilitation use.

## Figures and Tables

**Table 1:** Operational definition of MVC-normalized force-level labels.

Label	Approximate MVC Level	Interpretation in This Study
2	10% MVC	Low force-level condition
3	30% MVC	Low-to-moderate force-level condition
4	60% MVC	Moderate-to-high force-level condition
5	90% MVC	High force-level condition

**Table 2:** Predefined 26-feature set used for the main analysis.

Source	Feature Group	Feature Variables
sEMG	Time-domain amplitude and complexity	EMG_RMS, EMG_WL, EMG_ZC, EMG_SSC, EMG_WAMP
sEMG	Frequency and autoregressive features	EMG_MNF, EMG_AR1–EMG_AR4
MMG	Time- and frequency-domain features	MMG_RMS_Mech, MMG_Kurtosis, MMG_MPF
MMG	Cepstral-domain features	MMG_MFCC_1–MMG_MFCC_12
Fusion	Correlation-based coupling feature	Fusion_Corr_MA

**Table 3:** Feature sets used in the ablation analysis.

Feature Set	Number of Features	Included Information
sEMG-only	10	sEMG time-domain, frequency-domain, and AR features
MMG-only	15	MMG time-domain, frequency-domain, and MFCC features
sEMG+MMG	25	Direct concatenation of sEMG-only and MMG-only features
sEMG+MMG+Fusion_Corr_MA	26	sEMG+MMG features plus Fusion_Corr_MA

**Table 4:** Dataset structure used for model development and validation.

Item	Value
Window-level samples used for analysis	12,231
Action-trial groups	30
Force-level labels	2, 3, 4, and 5
Label 2 samples	2825 (23.10%)
Label 3 samples	2387 (19.52%)
Label 4 samples	3403 (27.82%)
Label 5 samples	3616 (29.56%)
sEMG-derived features	10
MMG-derived features	15
Correlation-based fusion feature	1
Predefined feature set used in main analysis	26 features
IMU-derived features included in main analysis	No

**Table 5:** Performance comparison of eight classifiers using the predefined 26-feature fusion set.

Classifier	Accuracy	Balanced Accuracy	Macro-F1	QWK	MAE
LR	0.797±0.033	0.814±0.026	0.812±0.026	0.812±0.055	0.290±0.060
KNN	0.709±0.027	0.737±0.018	0.730±0.020	0.648±0.049	0.467±0.046
DT	0.834±0.021	0.846±0.014	0.845±0.015	0.830±0.037	0.246±0.036
SVM-RBF	0.845±0.030	0.859±0.024	0.858±0.024	0.848±0.050	0.224±0.053
RF	0.886±0.023	0.897±0.017	0.895±0.018	0.883±0.042	0.169±0.044
ET	0.879±0.028	0.891±0.021	0.889±0.023	0.879±0.047	0.178±0.051
HGBDT	0.904±0.023	0.912±0.017	0.911±0.018	0.910±0.038	0.137±0.042
MLP	0.859±0.028	0.871±0.023	0.870±0.022	0.866±0.045	0.201±0.048

**Table 6:** Feature-set ablation analysis using HGBDT under group-aware cross-validation at the action-trial level.

Feature Set	Features	Accuracy	Balanced Accuracy	Macro-F1	QWK	MAE
sEMG-only	10	0.810±0.038	0.823±0.029	0.821±0.030	0.783±0.049	0.304±0.060
MMG-only	15	0.751±0.023	0.775±0.014	0.771±0.015	0.756±0.039	0.358±0.039
sEMG+MMG	25	0.904±0.023	0.912±0.018	0.911±0.019	0.910±0.037	0.138±0.041
sEMG+MMG+Fusion_Corr_MA	26	0.904±0.023	0.912±0.017	0.911±0.018	0.910±0.038	0.137±0.042

**Table 7:** Per-class performance of the 26-feature HGBDT model based on out-of-fold predictions.

Label	Precision	Recall	F1-Score	Support
2	0.913	0.922	0.917	2825
3	0.997	0.995	0.996	2387
4	0.831	0.842	0.837	3403
5	0.902	0.884	0.893	3616
Macro average	0.911	0.911	0.911	12,231
Weighted average	0.903	0.903	0.903	12,231

## Data Availability

The data presented in this study are available from the corresponding author upon reasonable request. The data are not publicly available because they contain participant-level physiological signal records and consent-limited information.
